# Development and Feasibility of the Headache-Related Light and Sound Sensitivity Inventories in Youth

**DOI:** 10.3390/children8100861

**Published:** 2021-09-28

**Authors:** Megan Silvia, Allison M. Smith

**Affiliations:** 1 Department of Physical & Occupational Therapy, Boston Children’s Hospital, Boston, MA 02115, USA; megan.silvia@childrens.harvard.edu; 2 Division of Pain Medicine, Department of Anesthesiology, Perioperative and Pain Medicine, Boston Children’s Hospital, Boston, MA 02115, USA; 3 Division of Psychology, Department of Psychiatry, Harvard Medical School, Boston, MA 02115, USA

**Keywords:** pediatric pain, pediatric headache, chronic headache, light sensitivity, sound sensitivity, photophobia, phonophobia, measure development

## Abstract

Youth with chronic headache disorders often experience sensitivities to light and sound that trigger or exacerbate their headaches and contribute to functional disability. At present, there are no known validated measures for assessing these sensitivities and their impact on functioning in youth with chronic headaches. This pilot study sought to develop and assess the feasibility of measures of headache-related light and sounds sensitivities in youth with chronic headache disorders. The initial item pools were generated via an intensive literature review, an informal quality improvement project, and a panel of experts in chronic pain. Then, youth (*n* = 20) presenting for clinical evaluation of headaches completed the revised items as well as assessments of the measures’ feasibility and items’ understandability. A subset (*n* = 2) completed formal cognitive interviews as well. The resulting 20-item Headache-Related Light Sensitivity Inventory (HALSI) and 18-item Headache-Related Sound Sensitivity Inventory (HASSI) for youth assess headache-related sensory sensitivities, as well as related emotional and behavioral responses. Through the iterative incorporation of feedback, these measures appear to be feasible to administer and understandable tools for assessing light and sound sensitivity in youth with chronic headache disorders. Once they are empirically validated, they have the potential to serve as important tools for understanding the patient experience, developing interventions, and assessing treatment response.

## 1. Introduction

Pediatric headache is common, with about 60% of children and adolescents prone to headaches over at least a three-month period [1]. While prevalence rates vary substantially depending upon headache subtype (e.g., migraine, tension-type, mixed-type, new daily persistent, post-concussive), it is well-established that youth who experience chronic headache disorders also report significant disability across all domains (e.g., physical, social, emotional, academic) [2,3]. In addition to the financial costs [4], headaches present considerable costs to youth roles, routines, and participation in meaningful occupations.

Chronic headaches in adults are often accompanied by atypical sensitivities to light and sound [5]. These sensitivities, often referred to as photophobia and phonophobia, respectively, can be conceptualized as comorbid symptoms of and/or triggers for headaches. They typically occur in response to non-noxious stimuli and contribute greatly to the headache experience [6]. One proposed mechanism for the presence of these atypical sensory sensitivities in adult headache patients is central sensitization of the nervous system [7,8]. Importantly, recent evidence does support the presence of central sensitization in youth with chronic headaches [9,10].

Photophobia and phonophobia have been characterized as “subjective experiences that alter sensory perception”, involving “limbic system pathways that superimpose an emotional processing of discomfort”… [11] (p. 1677). Consistent with the biopsychosocial model of chronic pain [12,13], this suggests cognitive, emotional, and behavioral factors, such as fear and activity avoidance [14], may impact the headache experience. In fact, specific relationships between pain, fear, avoidance, and disability are explained in the Fear Avoidance Model (FAM) [15,16]. According to the FAM, when a pain-related stimulus is perceived as threatening, patients develop fears of the stimulus and of pain itself. The fear often precipitates escape or avoidance behavior. In turn, the patient may become more disabled as they attempt to avoid stimuli/environments that may trigger pain [17]. Relatedly, the Trigger Avoidance Model of Headaches (TAMH) [18] purports that avoidance may also increase the stimulus’ potency through sensitization. Notably, though, the TAMH has not yet been validated in pediatric samples.

Despite the demonstrated relevance of light and sound sensitivities in pediatric headache disorders, there are currently no known validated measures that directly assess these important constructs, or the fear and avoidance related to them. Diagnostic assessment of light and sound sensitivity in the context of headaches typically entails a single-item dichotomous question (e.g., “Are you sensitive to light/sound”?). This does not account for variability in sensitivity severity or presentation, nor does it assess subjective experiences of fear and escape/avoidance behaviors [19,20]. In fact, youth may have difficulty understanding the question in the absence of a discussion of their emotional and behavioral responses to headache pain and sensitivity. Further, dichotomous response options are less ideal for tracking improvement after intervention.

Comprehensive tools for the measurement of headache-related sensory sensitivities in youth are desperately needed. Such tools would allow for a more thorough assessment of the patient’s headache experience, including the fear and avoidance that may be specific to the sensory sensitivities. Currently, measures of fear and avoidance in youth with chronic pain may not fully reflect the experience of youth with headache. For instance, though validated separately in a sample of youth with chronic headaches, items on the Fear of Pain Questionnaire—Child report (FOPQ-C) [14] are primarily movement-based. As such, the FOPQ-C items may not capture the fear and avoidance patterns of youth with headache, who may be “more concerned about other dimensions of pain-related fear”, such as cognitive or academic demands and environmental stressors [14] (p. 43). Similarly, the Headache Triggers Sensitivity and Avoidance Questionnaire (HTSAQ) [18] and the Cogniphobia Scale for Headache Disorders (CS-HD) [21] both have sensitivity, fear, and avoidance content specific to headaches, but both have very few items pertaining to light and sound specifically. Importantly, both measures are only validated in adult samples.

Drawing upon the extant literature, existing measures of relevant constructs, and the theoretical models described above, the primary goal of this pilot study was to generate and examine the feasibility/understandability of two separate item pools for potential measures of headache-related sensory sensitivities (i.e., one each for light and sound) that are comprehensive and inclusive of emotional and behavioral responses. This manuscript details the preliminary measure development steps, in following best practice guidelines for developing and validating scales in health behavior research [22]. Information gathered from these initial steps will provide the foundation needed for empirical validation in future studies. 

## 2. Materials and Methods

### 2.1. Domain Identification and Item Development

#### 2.1.1. Item Pool Generation

The initial item pools for the measures of light and sound sensitivity were generated by deriving item content from currently published chronic pain assessment tools for both adults and children. These included the HTSAQ [18], the CS-HD [21], and the Tampa Scale for Kinesiophobia (TSK) [23], each for adults, as well as the Fear of Pain Questionnaire (FOPQ-C) [14] for youth. The HTSAQ assesses avoidance of and sensitivity to common headache triggers, including several triggers pertaining to vision and one related to sound. The CS-HD assesses cogniphobia, a specific fear and avoidance of cognitive exertion, which is believed to precipitate or exacerbate headache. The TSK assesses kinesiophobia, an excessive, irrational, and debilitating fear of physical movement and activity resulting from a feeling of vulnerability to painful injury or reinjury. The FOPQ-c assesses fear and avoidance behaviors in response to pain. 

#### 2.1.2. Initial Testing with Patients and Clinicians for Content Validity

The two initial sets of items were administered for clinical purposes to patients presenting with chronic headache disorders, as part of the standard occupational therapy clinical assessment protocol in the authors’ Intensive Interdisciplinary Pain Treatment (IIPT) program. Through a six-month quality improvement (QI) initiative, measure administrators (i.e., three occupational therapists within the authors’ IIPT) were encouraged to document their experience administering and utilizing the data from these measures. Open-ended questions prompted administrators to detail administrative challenges they encountered (if any). This feedback was integrated into revisions whenever at least two of the three providers encountered the same challenge. 

#### 2.1.3. Evaluation by Expert Panel for Content Validity

To establish face and content validity, a total of 19 clinicians and researchers who specialize in pediatric chronic pain were invited to participate in an expert panel to review the preliminary items for both measures, with 15 invitees returning completed surveys. The panel consisted of physicians (e.g., neurologists, rheumatologists, anesthesiologists), psychologists, and occupational therapists, each with pediatric pain expertise. Panel members represented seven different pediatric hospitals affiliated with academic medical centers across the United States. Expert panel members provided both quantitative ratings of each item’s importance/suitability for inclusion (ranging from 0 = not at all important to 4 = very important). The a priori criterion for determining which items would be retained and which would be revised/dropped was the item’s mean rating on this scale. Items within a mean rating less than 3.0 indicated the need for substantial revision or elimination. The panel also provided qualitative feedback on individual items and the measures collectively. Specifically, they were prompted to provide suggested modifications to individual items and to offer additional items/concepts for inclusion in either measure.

### 2.2. Feasibility Assessment by the Target Population

#### 2.2.1. Participants

Twenty youth (M age = 15.8 years, SD = 1.99 years, range: 11–18 years; 100% female, 85% white) participated in the feasibility assessment. Participants’ diagnoses included new daily persistent headache (NDPH; 35%), migraine headaches (20%), tension-type headaches (10%), mixed-type headaches (25%), and post-concussive headaches (10%). Participants reported a mean pain duration of 47.15 months (i.e., nearly four years; SD = 26.08 months). They reported an average headache pain rating of 5.25 (SD = 2.02) on a 0–10 numerical rating scale, where 0 = no pain and 10 = the worst pain imaginable. 

#### 2.2.2. Procedure

The study was approved by Boston Children’s Hospital’s Institutional Review Board (IRB-P00033505 and IRB-P00039534). Participants were prospectively recruited during an initial evaluation of their chronic headache disorder at two sites within a large, urban pediatric hospital: (1) a multidisciplinary outpatient headache clinic; and (2) an IIPT. Participants were eligible to participate in the study if they held a primary diagnosis of a chronic headache disorder and were fluent in the English language. The only exclusionary criterion was having a moderate-severe developmental or cognitive delay that would render the task too difficult.

Informed consent for youth participation if under age 18 was provided by one caregiver; youth provided their assent/consent as relevant to their age. Once caregiver consent and youth assent were both obtained, participants first completed the measures of light sensitivity and sound sensitivity, in this order, with the administrator documenting the time it took the participant to complete each measure. Then, participants completed a standardized assessment of feasibility/understandability (developed specifically for this study) for each of the measures, administered in the same order. Next to each item, participants reported whether the following four issues occurred while completing the item, checking the box to indicate an affirmative response:

- This item doesn’t make sense to me.
- This item has a word I don’t understand (please circle that word).
- This item doesn’t fit in with the other items.
- This item bothers me or makes me mad.

The a priori criterion for determining which items were deemed understandable was the percentage of the sample endorsing one of the above issues with the item. If no more than 10% encountered a particular problem with the item, the item was deemed to be understandable and acceptable. Participants were also prompted to document item-specific feedback. Then, at the end of each feasibility questionnaire, participants were invited to respond to the prompt, “Is there anything else you would like us to know about the way light/sound affects your headaches that you think we have missed? Write it below”. Comments were grouped by theme where appropriate. When more than 10% of the sample (three or more participants) offered the same/similar suggestion(s), the change was incorporated into the measure.

#### 2.2.3. Pre-Testing via Cognitive Interviews

A subset of the sample (*n* = 2) were randomly selected to complete a cognitive interview about the measures. Because these participants had already met criteria for participation in the feasibility assessment, they were known to have the characteristics of interest for the interviews with regard to demographics and diagnostic status. Cognitive interviews took place individually in a quiet location, free from distraction, with the interviewer recording the participants’ responses. During the cognitive interviews, participants were asked to (1) read each test item; (2) describe aloud what they understand the question to mean for them; (3) explain their response aloud; and (4) offer open-ended feedback on the test item. The questions were phrased as follows: “What does this question mean in your own words? How did you come up with your answer? Is there anything you would change about this question?” Notes were taken throughout the interviews by the interviewer, including both verbal responses and observations. Interviews lasted 60 min and were conducted in English. Feedback was analyzed by the authors in conjunction with the feedback provided on the feasibility assessment.

## 3. Results

### 3.1. Item Pool Generation and Initial Testing

From the literature and clinical experience, the authors generated an initial item pool of 15 light sensitivity items and 12 sound sensitivity items for the measures. The measure format was initially a dichotomous response choice (e.g., yes/no). However, feedback from initial testing within the IIPT by all three clinicians (100%) indicated that a Likert-type response format would instead be most suitable for the items. Additionally, suggestions from the initial testing (as well as continued literature review) expanded both measures considerably, resulting in a 23-item light sensitivity item pool and a 20-item sound sensitivity item pool. Individual item structure was similar across both measures, but content was specific to the presenting concern. Upon examination of the items generated at this point, the authors proposed that for both measures, items would cover three content domains: sensitivity symptoms, emotional responses to the sensitivity, and behavioral responses to the sensitivity. These hypothesized domains will be statistically examined in a future study and are used here only for organizational purposes.

### 3.2. Expert Panel

#### 3.2.1. Quantitative Analysis

Based on the feedback from the expert panel, the 20 items in the original sound sensitivity item pool had a mean suitability rating of 3.33 (SD = 0.47) on the 5-point Likert scale (ranging from 0–4). There were five items with a mean rating that was <3.0, suggesting the need for substantial revision or elimination. Of these five, three were revised and two were eliminated. Similarly, the 23 items in the original light sensitivity item pool had a mean suitability rating of 3.44 (SD = 0.45) on the 0–4 scale. There were three items with a mean rating <3.0. Of these three, one item was revised and two were eliminated. Of note, the eliminated items were parallel across both item pools: “It’s not really safe for a person with a headache condition like mine to [see bright lights/hear loud sounds]” and “No one should be exposed to [bright lights/loud sounds] when they are experiencing headache pain”. In the comments, most panelists suggested removing both items due to questionable relevance/coherence with the other items (e.g., the first item due to its reference to subjective safety, the second because it assessed attitude).

#### 3.2.2. Qualitative Analysis

Thematically, many panelists’ suggestions, particularly from physicians, led to the reconceptualizing the measures not as an assessment of “photophobia” and “phonophobia”, but rather as headache-related light and sound sensitivity, given the specific neurological connotation of photo/phonophobia. Panelists also highlighted the variability in possible sensitivity presentation, most notably the likely scenario of a patient experiencing heightened sensitivity to typically benign sensory stimuli. Thus, the authors replaced descriptors such as “loud [sounds]” and “bright [lights]” with “certain [sounds/lighting]”. Panelists’ suggestions focused on the literacy level of the items, such as replacing “modify” with “change”.

The panelists also offered a total of 11 new items for the sound sensitivity item pool and 8 new items for the light sensitivity item pool. New items were either added in their own right, replaced a low-rated item, or were incorporated into existing items, depending on the existing content. Many new items pertained to the avoidance of stimuli or environments with certain sounds or lighting. Lastly, panelists suggested eliminating “neutral” from the response options, as the original version was a five-point Likert-type scale. This forced choice ensured that the most potential information was gleaned from each item.

### 3.3. Feasibility Assessment

Participants’ responses yielded generally positive feedback on domain content. No more than 10% (*n* = 2) of the sample endorsed having any of the feasibility/understandability issues (described in Section 2.2.2) for any item (e.g., doesn’t make sense, doesn’t fit with the others, bothers me/makes me mad). Additionally, only one participant identified a word they did not understand within an item (i.e., “fluorescent” in reference to lighting).

The open-ended responses and item-specific comments provided by participants also offered valuable feedback about item redundancy, ideas for item expansion, necessary wording clarifications, and changes to response options. Several participants (*n* = 5) highlighted the importance of differentiating between two school-oriented items in each item pool to reduce redundancy. Other participants made cogent suggestions for additions to existing items, such as incorporating other screened devices (e.g., tablets, videogaming screens, smartboards) to items about dimming screens (*n* = 3) or adding “closing the curtains/shades” to items about reducing incoming light (*n* = 3). Many participants offered consistent re-wording suggestions, such as replacing “afraid, nervous, or scared” with “worried” (*n* = 3) and replacing “cranky” with “upset” (*n* = 3).

Structurally, the initial items had been placed on a four-point Likert-type scale, ranging from 0 (strongly disagree) to 3 (strongly agree). However, participants (*n* = 5) suggested that items were clearer when the response anchors of the scale ranged from 0 (never) to 3 (always). Finally, the average time to complete the items was less than three minutes each (light sensitivity: 2 min, 27 s; sound sensitivity: 2 min, 42 s).

### 3.4. Cognitive Interviews

Cognitive interview participants were able to accurately describe the items in both pools in their own words to demonstrate good comprehension of each item. Themes for potential revisions were generally consistent with revisions suggested in the other phases of measure development. Regarding the measure framework, both participants in the cognitive interviews suggested removing the phrase, “In the last seven days”, from the directions in order to allow participants with episodic headache presentations to accurately reflect their experiences. They also reiterated the suggestion described earlier to use anchors ranging from “never” to “always”, in place of “strongly disagree” to “strongly agree”. At the item level, participants suggested reviewing school-related items for clarity about which aspects of the school experience were being addressed and merging similar school-related items to avoid redundancy in the measure. Interestingly, participants recommended rewording items beginning with “I can’t”… to “I don’t”… to reflect the distinction between participants’ physical capability versus performance. All suggested revisions were considered by the authors and incorporated as appropriate. The principal investigator (MS) made the final decision when consensus was not reached.

### 3.5. Resulting Measures

#### 3.5.1. Light Sensitivity

The proposed 20-item pool for the Headache-Related Light Sensitivity Scale (HALSI) is intended to constitute a patient-report measure that assesses headache-related sensitivity to light. All items ask the participant to respond to each item on a 4-point Likert-type scale, ranging from 0 (never) to 3 (always), with higher ratings indicating greater sensitivity to light. The 20-item item pool had a Cronbach’s alpha of 0.9. Examination of item-total statistics indicated no remarkable improvement in internal consistency (i.e., Cronbach’s alpha did not increase) with the removal of any item. Corrected item-total correlations ranged from 0.20 to 0.87. The sample mean total score for the pool items was 31.8 (SD = 9.96), with a range of 18–58 (out of a possible 0–60). Total scores were normally distributed across the sample. Table 1 provides a subset of the proposed HALSI items retained in this measure development process.

#### 3.5.2. Sound Sensitivity

Similarly, the proposed 18-item pool for the Headache-Related Sound Sensitivity Inventory (HASSI) is intended to constitute a patient-report measure that assesses headache-related sensitivity to sound. All items ask the patient to respond to each item on a 4-point Likert-type scale, ranging from 0 (never) to 3 (always), with higher ratings indicating greater sensitivity to sound. This 18-item item pool had a Cronbach’s alpha of 0.9. Examination of item-total statistics indicated no improvement in internal consistency (i.e., Cronbach’s alpha did not increase) with the removal of any item. Corrected item-total correlations ranged from 0.49 to 0.88. The sample mean total score for the pool items was 26.30 (SD = 12.44), with a sample range of 0–54, which represents to full possible range. Total scores were normally distributed across the sample. Table 2 provides a subset of the proposed HASSI items retained in this measure development process.

## 4. Discussion

A multi-step measure development process following best practice guidelines [22] was utilized in order to generate two comprehensive item pools to assess headache-related light and sound sensitivities in youth with chronic headaches. The resulting item pools, tentatively named the HALSI and the HASSI, are designed to assess not only the headache-related sensory sensitivity itself, but also the related emotional and behavioral responses to that sensitivity. The general item structure, Likert response type, and scoring is parallel across both pools, with content specific to light or sound as appropriate. Beyond what was known from the literature, participants at all stages (e.g., initial testing, expert panel, feasibility study, cognitive interviews) spontaneously indicated the clinical need for measures of this nature when providing their feedback.

Overall, feedback from the initial testing and the expert panel helped to expand the item pool to contain critical features of and behaviors associated with the light/sound sensitivities. The panel also provided valuable feedback about the response pattern/choices in order to glean the most from the data obtained. Then, feedback from youth participants helped to refine the measures for clarity and readability and to eliminate perceived redundancy. Statistically, the item pools were both internally consistent, with reasonable item-total correlations. Participants could accurately describe all items in their own words and demonstrated a good understanding of each item. Importantly, the questionnaires were completed in less than three minutes each, suggesting that administration in the clinic setting is feasible. Each phase of the process was intentionally iterative, ensuring that all stakeholders’ perspectives were considered.

The findings of this preparatory study must be viewed in light of its limitations. The sample size was small, though typical for initial piloting and feasibility exploration [22], and homogenous demographically, consisting predominantly of white females. However, this is generally reflective of the demographic characteristics of pediatric headache patients [24]. Relatedly, the sample size was somewhat small for cognitive interviews [25] and conducted as a single round. However, for both the feasibility questionnaire and the cognitive interviews, responses were notably consistent across participants. Therefore, data collection continued until relatively few new insights emerged, which is considered the ideal approach for studies of this kind [22,25].

The preliminary development of the HALSI and HASSI item pools and findings from this feasibility study have several implications for future research endeavors among youth with chronic headaches. First, and most directly, these findings have provided the necessary foundation for the ensuing steps in scale development, including an examination of item performance and factor analysis to explore the structure and internal consistency of each measure. As such, even after the initial measure validation, additional studies may be necessary to further optimize and support the proposed measures in samples of youth experiencing chronic headaches. Further, these findings may also inform future interventions for children with chronic headaches. For instance, additional research using these measures (once validated) may further elucidate the role of fear and avoidance of light/sound as intervention targets for youth with chronic headaches. This understanding may help to delineate appropriate and effective therapeutic strategies for facilitating functional improvement, particularly in patients who avoid activities and environments due to their light and sound sensitivities.

Overall, based on the initial steps of measure development completed in this study, the HALSI and HASSI item pools appear to contain feasible and understandable items for assessing light sensitivity and sound sensitivity among youth with chronic headache conditions. Once their item performance and factor structure are validated, their use may provide valuable insights for clinicians and researchers seeking to understand not only light and sound sensitivities themselves, but their emotional and behavioral components that may impact functioning. This study has provided the foundation for the creation of important measures for intervention development and assessing treatment response in IIPT and other treatment settings.

## Figures and Tables

**Table 1:** Subset of items for proposed inclusion in the HALSI.

Example HALSI Item	Hypothetical Scale ^1^
Certain lighting (such as bright light, sunlight, fluorescent light, or flashing light) triggers my headache or makes it worse	Sensitivity Symptom
I dim or lower the brightness on electronic screens (such as computers, TVs, phones, tablets, or gaming devices) because of my headache	Behavioral Response
I block out lighting that affects my headache by wearing hats, visors, or sunglasses, or covering my eyes in some other way	Behavioral Response
Because of the way lighting affects my headache, I avoid or change the way I participate in social or fun activities like going to the movies, concerts, or arcades	Behavioral Response
I worry that certain lighting might trigger my headache or make it worse	Emotional Response
Because of my headache, certain lighting makes me feel stressed or upset	Emotional Response

^1^ Scales are hypothetical, based only on the literature, and are to be explored in future studies.

**Table 2:** Subset of items for proposed inclusion in the HASSI.

Example Item	Hypothetical Scale ^1^
Certain sounds (such as loud sounds, high-pitched sounds, or many sounds at once) trigger my headache or make it worse	Sensitivity Symptom
I ask people around me to be quiet or turn down the volume because of my headache	Behavioral Response
I block out sounds that affect my headache by wearing headphones or covering my ears in some other way	Behavioral Response
Because of the way sounds affect my headache, I avoid certain places in my school (other than the classroom), such as the cafeteria, hallways, or gymnasium	Behavioral Response
I worry that certain sounds might trigger my headache or make it worse	Emotional Response
Because of my headache, certain sounds make me feel cranky or annoyed	Emotional Response

^1^ Scales are hypothetical, based only on the literature, and are to be explored in future studies.

## Data Availability

The data presented in this study are available on request from the corresponding author. The data are not publicly available due to the privacy policies in place at the institution at which the data was collected.
